# *De novo* assembly and analysis of the transcriptome of the *Dermacentor marginatus* genes differentially expressed after blood-feeding and long-term starvation

**DOI:** 10.1186/s13071-020-04442-2

**Published:** 2020-11-10

**Authors:** Ercha Hu, Yuan Meng, Ying Ma, Ruiqi Song, Zhengxiang Hu, Min Li, Yunwei Hao, Xinli Fan, Liting Wei, Shilong Fan, Songqin Chen, Xuejie Zhai, Yongchang Li, Wei Zhang, Yang Zhang, Qingyong Guo, Chahan Bayin

**Affiliations:** 1 College of Animal Science, Xinjiang Agricultural University, Ürümqi, 830052 Xinjiang Uygur Autonomous Region People’s Republic of China; 2 College of Veterinary Medicine, Xinjiang Agricultural University, Ürümqi, 830052 Xinjiang Uygur Autonomous Region People’s Republic of China; 3 College of Animal Science and Technology, Qingdao Agricultural University, Qingdao, 266109 Shandong Province People’s Republic of China; 4 National Research Center for Protozoan Diseases, Obihiro University of Agriculture and Veterinary Medicine, Obihiro, Hokkaido 080-8555 Japan; 5 Bayingol Vocational and Technical College, Korla, 841000 Xinjiang Uygur Autonomous Region People’s Republic of China

**Keywords:** *Dermacentor marginatus*, RNA-seq, Transcriptomics, Tick physiology, Differential expression

## Abstract

**Background:**

The ixodid tick *Dermacentor marginatus* is a vector of many pathogens wide spread in Eurasia. Studies of gene sequence on many tick species have greatly increased the information on tick protective antigen which might have the potential to function as effective vaccine candidates or drug targets for eco-friendly acaricide development. In the current study, RNA-seq was applied to identify *D. marginatus* sequences and analyze differentially expressed unigenes.

**Methods:**

To obtain a broader picture of gene sequences and changes in expression level, RNA-seq was performed to obtain the whole-body transcriptome data of *D. marginatus* adult female ticks after engorgement and long-term starvation. Subsequently, the real-time quantitative PCR (RT-qPCR) was applied to validate the RNA-seq data.

**Results:**

RNA-seq produced 30,251 unigenes, of which 32% were annotated. Gene expression was compared among groups that differed by status as newly molted, starved and engorged female adult ticks. Nearly one third of the unigenes in each group were differentially expressed compared to the other two groups, and the most numerous were genes encoding proteins involved in catalytic and binding activities and apoptosis. Selected up-regulated differentially expressed genes in each group were associated to protein, lipids, carbohydrate and chitin metabolism. Blood-feeding and long-term starvation also caused genes differentially expressed in the defense response and antioxidant response. RT-qPCR results indicated 6 differentially expressed transcripts showed similar trends in expression changes with RNA-seq results confirming that the gene expression profiles in transcriptome data is in consistent with RT-qPCR validation.

**Conclusions:**

Obtaining the sequence information of *D. marginatus* and characterizing the expression pattern of the genes involved in blood-feeding and during starvation would be helpful in understanding molecular physiology of *D. marginatus* and provides data for anti-tick vaccine and drug development for controlling the tick.
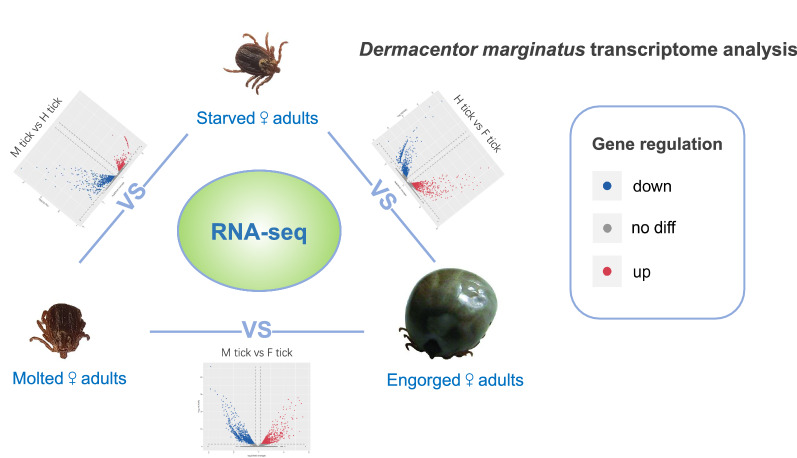

## Background

Ticks are obligate hematophagous ectoparasites of great medical and veterinary importance [1]. *Dermacentor marginatus* Sulzer that is a wide spread tick species of the Ixodidae carries and transmits a variety of tick-borne pathogens to livestock and human, such as *Babesia* [2], *Theileria* [3], *Anaplasma* [4], *Rickettsia* [5], *Coxiella* [6], *Brucella* [7], *Borrelia burgdorferi* [8] and Orthonairovirus [9]. Due to its medical importance, *D. marginatus* as many other tick species, has become an emerging threat to the livestock and public health, which needs control measures to mitigate its impact.

Currently, the accessibility and ease of practice have made chemical approach dominant in tick control measures [10]. While the use of conventional acaricides is effective in tick control, the environment is affected by contamination and acaricide residue problems [11]. Furthermore, tick resistance to acaricide has made chemical agents less effective than before [12]. Thus, finding the important target genes of tick species is the key to develop advanced tick control approaches [13, 14].

The research on tick prevention and control methods is greatly facilitated by revealing genome data of tick species. The genome data of *Ixodes scapularis* Say is the first genomic functional study that provided abundant references to gene annotations of tick species [15]. A very recent study on six tick genomes expounded the comparative genome analyses of ixodid tick species [16]. Together with the *I. scapularis* genome data, these tick genome data could be of crucial values for reference templates for omics studies such as transcriptome and proteome analyses on ixodid ticks and other tick species [16]. Currently RNA-seq is an effective approach to obtain transcripts on a specific time point. Many transcriptome studies revealed a large number of tick transcripts that were latter used for tick gene function study [17–20], system evolution study [21], and anti-tick vaccine candidate screening [22, 23].

Vaccination of livestock is a promising method considering labor-saving, cost-effective for farmers and an eco-friendly approach for mitigation of ticks [24]. But only one available tick vaccine against Bm 86 of *Rhipicephalus microplus* Canestrini is available, which is less effective against ticks other than *R. microplus* [25, 26]. New tick-specific antigens are still in need. Now screening of potential protective tick antigens could be achieved by RNA-seq and tick vaccine trials [23]. On the other hand, researches focusing on biological process of tick blood-feeding and reproduction has enhanced our knowledge on the functions of important tick molecules [27, 28]. According to the study of tick blood meal digestion, Ixodidae ticks has a different way of blood digestion from other hematophagous arthropods by intracellular digestion of hemoglobin [29]. In the process of blood-feeding and digestion of blood meal, there were many genes upregulated encoding proteins, such as ferritin 2 transporting ferric iron in the hemolymph [30], aquaporin regulating body fluid and facilitate salivation [31], and vitellogenin and vitellogenin receptor critical for embryonic genesis [32]. These tick antigens showed potential to compose an anti-tick vaccine.

Besides feeding on hosts, hard ticks live off-host for most of their lives. Ticks conduct sit-and-wait strategy in questing with an extreme endurance to starvation [18]. Ticks initiate different set of genes to cope with long-term starvation and their questing behavior will increase in extended starvation times [18]. During non-feeding period many tick species may express crucial protein for survival that in turn can be harnessed as targets for chemicals. In addition, development of eco-friendly acaricides or tick repellents is also an promising field of tick control [33]. Reports indicated that new acaricide target genes could be used to develop effective but environmentally friendly chemicals to control tick prevalence, for instance, tick kinin receptors regulate numerous tick physiological process including ecdysis and feeding [34].

Regarding *D. marginatus*, the study was in the hope of enriching the molecular information for this tick species and provide a comprehensive viewpoint of this biological system, as current information on *D. marginatus* is limited. Our focus was on three different status of *D. marginatus* female adults as newly molted, starved, and engorged state to conduct an Illumina RNA sequencing (RNA-seq) in order to obtain the transcriptomes of *D. marginatus* female adults. This study of whole tick transcriptome could contribute to understanding of genes involved in blood-feeding and long-term starvation.

## Methods

### Tick rearing

Ticks used in the experiment were F2 generation progeny of a *D. marginatus* adult female originally collected from a 6-year old local-bred female horse in a breeding farm located in Yili prefecture, Xinjiang, China, in April 2018. Ticks were reared under controlled experimental conditions of temperature (24 ± 1 °C), relative humidity (RH; 90 ± 5%) and photoperiod (14 h light: 10 h dark). The purpose of the RNA-seq analysis was to obtain gene sequences and identify differentially expressed genes of *D. marginatus* female adults after blood-feeding and long-term starvation regarding the significant role of female adult that plays in feeding large amount of blood and producing thousands of eggs per individual [35]. Another reason choosing females only for the RNA-seq analysis was to minimize the gene expression difference profiled by sexual distinction [36, 37].

**Fig. 1:**
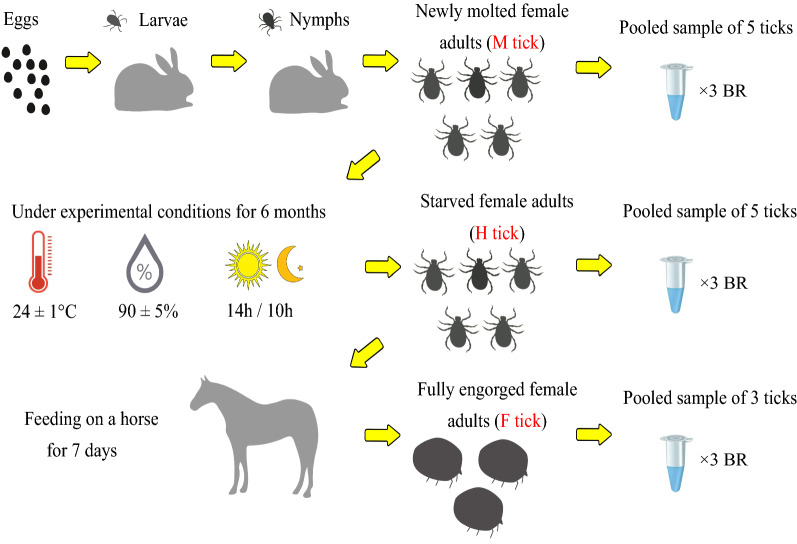
Schematic demonstration of the experimental design. F2 generation larvae hatched from a single egg clutch. Larvae and nymphs were fed on two experimental rabbits. Five newly molted female adult ticks (M tick group) were pooled as one sample and three biological replicates (BR) were prepared. Batches of ticks were placed under controlled experimental condition for 6 months to reach long-term starved condition (H tick group). Among the ticks, five female adult ticks were pooled as one sample and three biological replicates were prepared. The long-term starved ticks were fed on horse blood to become engorged, and manually removed on 8th day. The engorged female adult ticks were place under controlled experimental condition for 24 h before sample preparation (F tick group). Three engorged female ticks were pooled as one sample and 3 biological replicates were prepared. RNA was extracted at each time point and mode of state is represented by three independent libraries for each group. In total, the RNA-seq is based on nine pooled whole-tick libraries

In order to obtain the *D. marginatus* female adults, we designed three biological groups for RNA-seq analysis (Fig. 1). Larvae (*n* ≈ 1000) and nymphs (*n* = 400) fed on the ears of two New Zealand rabbits respectively in succession to become engorged. Subsequently, 363 engorged nymphs successfully molted to adult stage. Adult female ticks after ecdysis for 5 days were designated as M tick group that consisted of 3 biological replicates each made of 5 ticks. Female adult ticks were kept under controlled experimental conditions (as above) for 6 months to become starved ticks as H tick group that consisted of 3 biological replicates each made of 5 ticks. Finally, F tick group that consisted of 3 biological replicates each made of 3 engorged female adult ticks was prepared by having 30 starved female adult ticks fed on a local bred yearling for 7 days with 60 male adult ticks around (Fig. 1). The engorged female adult ticks were carefully removed on the 8th day after blood-feeding and kept at controlled experimental conditions (as above) for 24 h before sample preparation started.

### Preparation of the biological samples and total RNA extraction

Body weight of similar *D. marginatus* female adults were selected to make pooled samples. Sample were prepared as fast as possible to reduce human factors on gene expression. To clean the surface of the ticks, we washed the ticks in phosphate buffer saline (PBS) though mild shaking for 15 s in the first round. Washing the ticks in 70% alcohol by the same operation followed next. Last round, wash the ticks in deionized water though mildly shaking for 30 s. Then, after drying the ticks on sterile gauze, ticks that were put in microtubes were rapidly immersed into liquid nitrogen to freeze completely and stored at – 80 °C for later use.

Total RNA was extracted using Trizol reagent (Invitrogen, Carlsbad, CA, USA) following the manufacturer’s procedure. The total RNA quantity and purity were analyzed with Bioanalyzer 2100 (Agilent, Foster, CA, USA) and RNA 1000 Nano LabChip Kit (Nanodrop Technologies, Wilmington, DE, USA) with RIN number > 7.0. Poly (A) RNA is purified from total RNA (5 μg) using poly-T oligo-attached magnetic beads using two rounds of purification.

### The cDNA library preparation

After purification, the mRNA was fragmented into small pieces with divalent cations under elevated temperature. Then the cleaved RNA fragments were reverse-transcribed to create the final cDNA library in accordance with the protocol for the mRNA Seq sample preparation kit (Illumina, San Diego, CA, USA), the average size of the insert for the paired-end libraries was 300 bp (± 50 bp). Then we performed the paired-end sequencing on an Illumina Hi-seq 4000 (LC Sciences, USA) following the vendor’s recommended protocol.

### *De novo* assembly, unigene annotation and functional classification

Firstly, Cutadapt [38] and in-house perl scripts were used to remove the reads that contained adaptor contamination, low quality bases and undetermined bases. Then sequence quality was verified using FastQC (http://www.bioinformatics.babraham.ac.uk/projects/fastqc/) including the Q20, Q30 and GC-content of the clean data. All downstream analyses were based on clean data of high quality. *De novo* assembly of the transcriptome was performed with Trinity v. 2.4.0 [39]. Trinity groups transcripts into clusters based on shared sequence content. The longest transcript in the cluster was chosen as the unigene sequence.

The prediction of coding sequences (> 100 amino acids) was performed using TransDecoder, which is part of the Trinity software [40]. All assembled unigenes were aligned against the non-redundant (Nr) protein database (http://www.ncbi.nlm.nih.gov/), Gene ontology (GO) (http://www.geneontology.org), SwissProt (http://www.expasy.ch/sprot/), Kyoto Encyclopedia of Genes and Genomes (KEGG) (http://www.genome.jp/kegg/), Protein family (Pfam) (http://pfam.xfam.org/) and Eggnog (http://eggnogdb.embl.de/) databases using DIAMOND [41] with a threshold of E value < 0.00001. Local BLAST v. 2.9.0 (ftp://ncbi.nlm.nih.gov/blast/executables/blast+/LATEST/) was used to compare the RNA-seq data of *D. marginatus* with genome sequencing data of *D. silvarum* [16]. Signal peptide prediction of the unigenes encoding proteins was performed with SignalP - 5.0 server (http://www.cbs.dtu.dk/services/SignalP/).

### Differentially expressed unigene analysis

Salmon [42] was used to perform expression level for unigenes by calculating transcripts per million (TPM) [43]. The differentially expressed unigenes were marked with log2 (fold change) > 1 or log2 (fold change) < − 1, and gene expression significance was based on Benjamini-Hochberg false discovery rate (FDR: *P* < 0.05) and one-way analysis of variance (one-way ANOVA: *P* < 0.05) by R package *edgeR* [44]. GO and KEGG enrichment analysis were again performed on the differentially expressed unigenes by in-house perl scripts.

### Real-time quantitative PCR validation of RNA-seq results

The consistency of the gene expression levels was confirmed by real-time quantitative PCR. The RT-qPCR was performed on another set of samples in a separate tick feeding experiment with the same sample preparation procedure above-mentioned. and the expression levels of 6 particular genes were assessed. Ferritin 1 (DN38021), Ferritin 2 (DN51538), HSP 70 (DN48195), galectin (DN57588), glutathione S-transferase (DN38169), and Dm86 (DN57937) were selected among the genes differentially expressed in newly molted (M tick), starved (H tick), and fed female ticks (F tick). RNA extraction procedure was performed as described above. The first strand cDNA templates were synthesized using a Fastking RT cDNA synthesis kit (Tiangen Biotech, Beijing, China). Single-strand cDNA was used, and transcription profile was conducted through real-time PCR using QuantiNova SYBR Green PCR Kit (Qiagen, Hilden, Germany) with an Applied Biosystems 7500 Fast Real-Time PCR System (Applied Biosystems, Foster, USA). Specific primers for target gene and the internal control are shown in Additional file 1: Table S1. The RT-qPCR procedure started with initial denaturation at 95 °C for 2 min, followed by 40 cycles of a denaturation step at 95 °C for 10 s and an annealing/extension step at 60 °C for 35 s. The mRNA levels of the analyzed genes both in transcriptome and RT-qPCR were normalized using the elongation factor-alpha 1 (*ef-α1*) gene of *D. marginatus* (DN43634) using the comparative Ct method [45].

## Results and discussion

### Sequencing and assembly

**Fig. 2:**
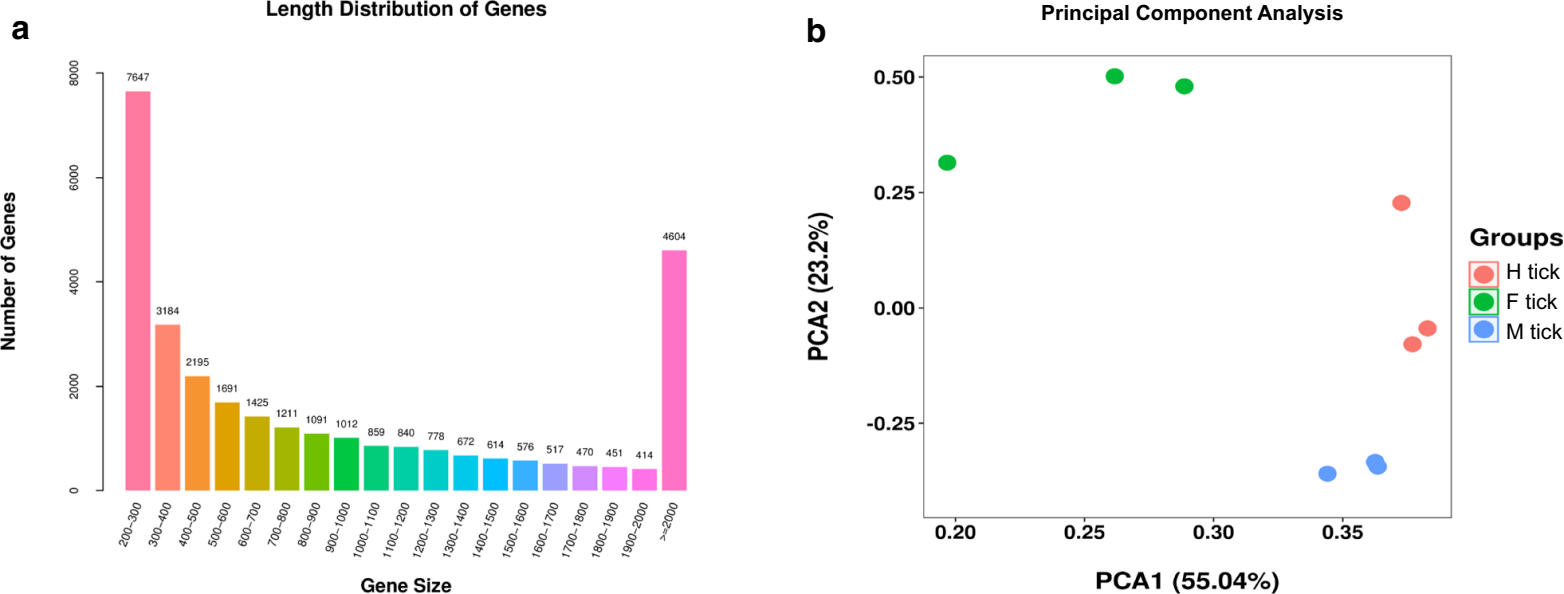
Summary of the assembled unigenes and PCA analysis of the libraries. **a** The X axis shows sequence length of the unigenes and Y axis is the number of unigenes. **b** The X axis represents first principal component and Y axis stands for second principal component. Red points represent libraries of H tick group. Green points represent libraries of F tick group and red points represent libraries of M tick group

Illumina sequencing of the nine *D. marginatus* adult female tick samples resulted in 460,171,414 raw reads (Additional file 2: Table S2). After removing low quality reads, 449,349,046 valid reads accounted for 97.68% of the raw reads. *De novo* transcriptome assembly was performed using Trinity software, resulting in a total of 100,644 putative transcripts (N50 1383), clustered into 30,251 unigenes. A summary of the assembly is shown in Table 1. Regarding sequence length, all unigenes are longer than 200 bp. The longest is 24,550 bp. Fifteen percent of assembled unigenes were above 2000 bp, and many of the unigenes were between 300–1000 bp (39.04%) (Fig. 2a). Principal components analysis (PCA) of the assembly revealed biological replicates grouped together separately showing acceptable variation within groups (Fig. 2b). It is notable that this is the first transcriptome analysis of *D. marginatus* produced using RNA-Seq. The assembly results of this study have augmented sequence information of *D. marginaus.* The transcriptome data used in this analysis were submitted to GEO (Gene Expression Omnibus, http://www.ncbi.nlm.nih.gov/geo/) database of NCBI under the accession number GSE151667.

**Table 1:** Summary of the *D. marginatus* transcriptome assembly

Index	All	Min length	Mid length	Max length	Total assembled bases	N50
Transcript	100,644	201	509	24,550	86,647,606	1383
Unigene	30,251	201	628	24,550	31,889,293	1822

### Annotation

**Fig. 3:**
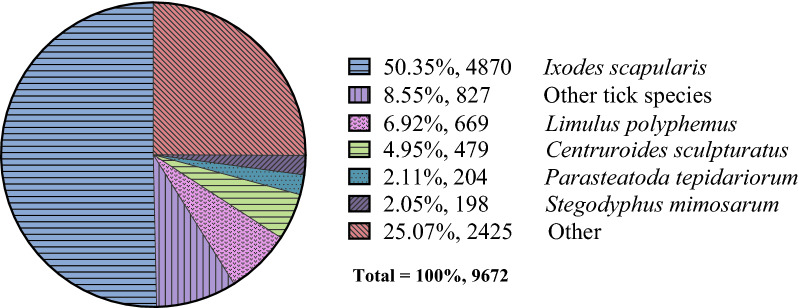
The pie chart of taxonomic assignment of unigenes. The percentage of unigenes assigned to each taxonomic group is shown on the right. Best hits to “Other” include arthropods other than tick species

Functional annotation of the assembled unigenes were performed by matching the 30,251 unigene sequences against 6 databases. Nr database hit the highest match up to 32%, while Swissprot database hit 22.56%, relatively less than the other database (Additional file 3: Table S3). Among the 9672 annotated unigenes, 58.9% of them showed homology with sequences of tick species, and over half of the unigenes matched *I. scapularis* sequences (Fig. 3). The remaining 20,579 unigenes (68.03%) were classified as unknown because they did not produce matches within the searched databases, indicating that many new genes and non-coding RNA sequences had been identified [37, 46]. Genome data is very important to tick transcriptome studies. Recently published tick genome study described 6 tick species that are common in the Old Continent [16]. These genome data are valuable in studying tick gene function, system evolution, and symbiotic relationship of the pathogen in different tick species. Among the genome data, *Dermacentor silvarum* Olenev is a closely related tick species with *D. marginatus*. The two tick species are morphologically similar, but surprisingly genomic alignments of the *D. marginatus* RNA-seq data with genome sequencing data of *D. silvarum* showed that the two tick species did not show very high sequence similarity (Additional file 4: Table S4). The reason that most of hits matched with *I. scapularis* rather than *D. silvarum* was probably because sequence information on *I. scapularis* is abundant in the searched databases currently [15]; nevertheless, the *D. silvarum* genome sequencing data would be soon available as reference for annotation in the databases. We performed a Local BLAST to compare the RNA-seq data of *D. marginatus* with genome sequencing data of *D. silvarum*, and the results revealed that 5841 out of 9573 unigenes with coding regions (CDs) matched that of genome sequencing data of *D. silvarum* with varying degrees. For identifying potential tick proteins, the SignalP prediction was performed and the results indicated that 320 out of 9573 unigenes encoding proteins have signal peptide sequence, while 150 were among the unidentified unigenes. The SignalP prediction results indicated that many potential tick genes encoding proteins are still waiting to be uncovered. The unigene annotation table, sequence alignment results and the signal peptide prediction results were incorporated as Additional file 5: Table S5, and the information contained in the table could be helpful in finding potential tick gene orthologs and proteins.

### Gene Ontology (GO) classification

**Fig. 4:**
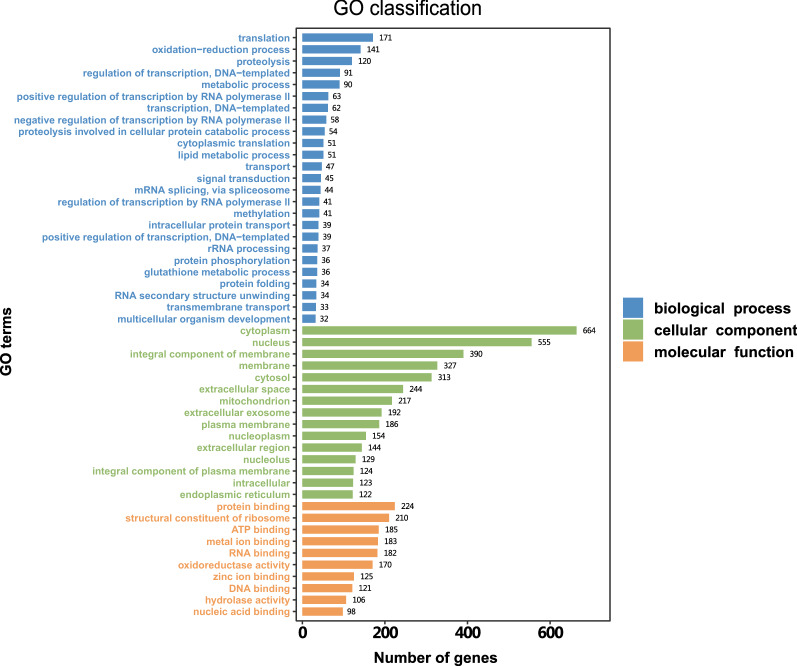
Gene Ontology classification of *D. marginatus* transcriptome data. Bars and numbers represent number of genes in each protein class. Blue bars refer to biological process, green bars refer to cellular component and orange bars refer to molecular function. The X axis shows the number of unigenes in each category

The annotated sequences were next functionally characterized using the information available in GO classification that considers 3 categories. Initially, genes were classified according to their molecular function, cellular component, and biological process using the GO terms from the GO database (Fig. 4). Only the categories represented by more than 30 genes are depicted, each including the number of the genes assigned.

The most abundantly annotated functions were binding activity, such as protein binding activity (*n* = 211), ATP binding activity (*n* = 185) and metal ion binding (*n* = 183) which coincided with transcriptomes of other ixodid tick species [47, 48]. The remaining annotated functions were as follows: structural constituent of ribosome (*n* = 210), RNA binding (*n* = 182), oxidoreductase activity (*n* = 170), zinc ion binding (*n* = 125), DNA binding (*n* = 121), hydrolase activity (*n* = 106), nucleic acid binding (*n* = 98).

Gene classification according to cellular component resulted in up to 15 categories represented by more than 100 genes. In the cellular component category, the most abundant GO term was cytoplasm, assigned to 17.1% (*n* = 664) of the total GO annotated unigenes. Many GO terms related to nucleus were abundant either (*n* = 555). Membrane (*n* = 327) and integral component of membrane structure (*n* = 390) followed next.

Gene classification based on biological process resulted in 25 categories. Notably, the most abundant (*n* = 171) was translation process followed by categories corresponding to oxidation-reduction process (*n* = 141), proteolysis (*n* = 120), regulation of transcription (*n* = 91), and metabolic process (*n* = 90). The remaining annotations of biological process that contained less unigenes were distributed into twenty categories. The GO terms for molecular function, cellular compartment and biological process assigned to all the genes annotated are showed in Additional file 6: Table S6.

### Differential gene expression profiles

**Fig. 5:**
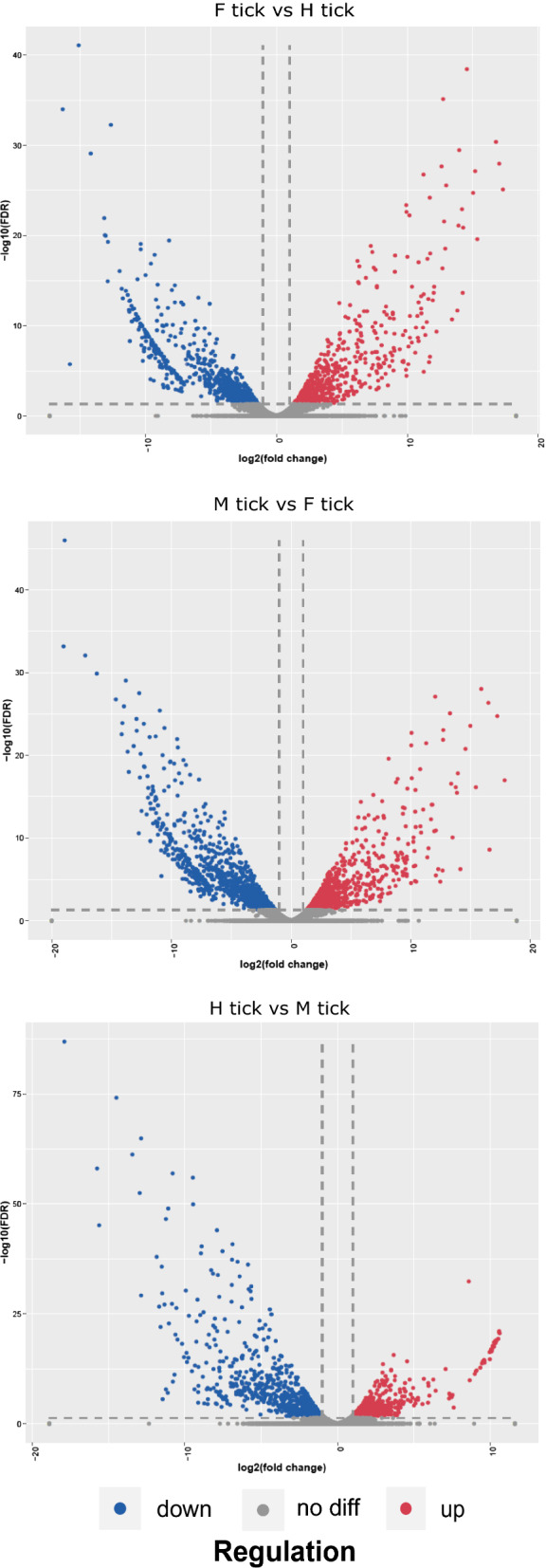
Scatter plots of the gene expression counts between groups. The X axis shows log2 fold-change that is plotted against the mean expression of each unigese of the two groups. Y axis is -log10 FDR value (Student’s t-test, *P* < 0.05) shown from 0 to 80. Every point represents one transcript. Differentially expressed transcripts having a fold change of > 1.5 and *P* < 0.05 (Student’s t-test) are represented by red and blue dots. Gray dots represent Genes not differentially expressed

Blood-feeding and long-term starvation caused significant differential gene expression of *D. marginatus*. The volcano plots were applied to demonstrate the overall gene expression profiles (Fig. 5). Differentially expressed genes (DEGs) were almost one third of the overall unigenes and the downregulated unigenes slightly outnumbered upregulated unigenes between each compared group (Additional file 7: Figure S1) indicating the three different states of *D. marginatus* female adults were experiencing significant physiological function shift which was in line with many ixodid transcriptome studies [18, 23, 49]. It is considered that H tick represents starved ticks that experienced 6 months non-feeding period exhausting its energy sources of lipid, carbohydrate, and protein, whereas M tick stands for newly molted ticks with a physiological status at its prime with many of the energy metabolism-related genes downregulated [18]. After mating, a *D. marginatus* female adult could ingest over 100-fold on its body weight in blood [35]. The blood meal is digested and the nutrients are transported and mostly used for embryogenesis [27]. Both blood-feeding and long-term starvation would cause significant differential gene expression.

**Fig. 6:**
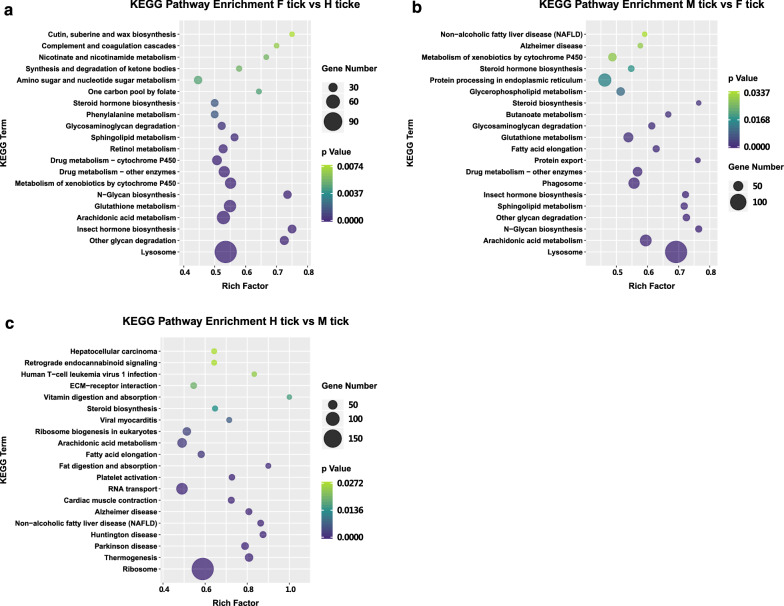
Significantly enriched KEGG pathways associated with blood-feeding and starvation. **a** Pathways enrichment between F tick and H tick. **b** Pathways enrichment between M tick and F tick. **c** Pathways enrichment between H tick and M tick. The top 20 most significant KEGG terms were illustrated for each compared pair. The X axis shows the rich factor that is the ratio of differentially expressed unigenes in a pathway term to all gene numbers annotated in the same pathway term. The bubbles represent the number of unigenes and the gradient coloration indicates the corrected *P*-value by the FDR method. Only pathways with *P* < 0.05 were shown

For further interpretation of systemic functions, the *D. marginatus* dataset was mapped to KEGG pathways. The 15 pathways most enriched in specific stages of female adult ticks are presented in Fig. 6. The significantly enriched pathways consisted of protein processing, glycan biosynthesis, lipid metabolism, detoxification, and defense responses.

Currently it is accepted that proteins in the blood meal are the main source of nutrient for oviposition and the energy source for resisting long-term starvation [18, 19, 50]. Upregulated genes in the three stages potentially involved in protein metabolism are listed in Table 2 and Additional file 5: Table S5. Protein families of proteases, such as cysteine peptidases, aspartic endopeptidases, metallopeptidases and serine peptidases were significantly upregulated in the F tick group (Fig. 6a) suggesting that the proteolytic system was active [46]. The results are quite similar to other transcriptome studies [22, 37]. The intracellular digestion of the proteins ingested with blood is performed by a group of lysosomal proteolytic enzymes that act sequentially and have been studied in detail in the family Ixodidae [27, 29, 51, 52]. It has been demonstrated that haemoglobin is the source of haem group for the development of the embryo in ixodids, and it is essential for a successful hatching of eggs spawned by engorged female ticks [22]. In the H tick group proteolysis enhanced, and pathways associated with diseases were underlined (Fig. 6c). In starvation, there was an upregulation of pathways related to transcription and translation processes that are energetically expensive. The result is in consistence with a previous study on *Dermacentor variabilis* Say [18]. It was probably caused by excessive consumption of nutrient substances so as to expend proteins in its cellular structure to maintain basic life support [18]. In the M tick group, pathways of steroid biosynthesis, protein export and N-Glycan biosynthesis were significantly enriched (Fig. 6b). New adult ticks emerging from engorged nymphs have consumed their last meal taken in their nymphal state. Host blood components, especially hemoglobin, were digested, and subsequently biosynthesized for new proteins acting on hormones and chitin biosynthesis [53]. As tick experience long-term starvation, the energy sustaining life comes mainly from consumption of proteins in tick midgut [18, 54, 55].

**Table 2:** Selected differentially expressed unigenes putatively involved in protein metabolism after blood-feeding and long-term starvation

Predicted Function	Annotation	TPM F-tick	TPM H-tick	TPM M-tick	ANOVA (*P-*value)	Unigene
Proteinase	Midgut cysteine proteinase	250.11	105.93	9.57	0.02	DN42765
	Hypothetical protein	244.25	0.71	0.01	0	DN53495
	Conserved hypothetical protein	0	69.91	14.74	0	DN35517
	Putative serine protease	58.94	84.12	16.04	0	DN46725
	Midgut cysteine proteinase 2	139.38	142.84	27.02	0	DN40266
	Astricsin	0	42.25	0	0	DN38987
	Conserved hypothetical protein	0.60	17.25	2.04	0	DN56731
Metalloprotease	Leucine aminopeptidase	125.81	21.76	9.79	0	DN46003
	Conserved hypothetical protein	233.58	60.91	36.54	0	DN58308
	Astacin-like metallopeptidase	10.37	2.76	0.60	0	DN37813
	Neprilysin	6.01	45.58	6.32	0	DN54652
	Astacin-like metalloprotease	8.29	0.05	0.04	0	DN23285
	Membrane metallo-endopeptidase	11.90	0.19	0.27	0	DN54193
	Zinc metalloprotease	6.17	26.69	2.60	0	DN56686
Cathepsin L	Cathepsin L-like cysteine proteinase	441.24	427.17	84.50	0	DN56942
	Cathepsin L	1,254.14	141.31	57.16	0	DN40829
Cathepsin B	Cathepsin B endopeptidase	522.98	106.56	12.00	0	DN54757
	Cathepsin B	65.82	46.24	11.38	0	DN52552
Cathepsin C	Cathepsin C precursor	148.74	222.46	47.97	0	DN42108
Cathepsin D	Yolk cathepsin	138.57	0.05	0.04	0	DN56458
	Aspartic protease	58.94	0.30	0.04	0	DN33194
	Heme-binding aspartic proteinase	146.34	0.11	0.05	0	DN36019
	Aspartic protease	35.25	127.92	10.05	0	DN44103
Carboxypeptidase	Carboxypeptidase	3.50	0.08	23.44	0	DN57960
	Conserved hypothetical protein	65.18	0.03	0.00	0	DN54491
Serine protease inhibitor	Serine protease inhibitor 3	199.21	9.24	16.00	0	DN39339
	Serine protease inhibitor 17	178.14	9.03	24.05	0	DN54400
	Serine protease inhibitor 12	27.37	3.68	44.21	0	DN53734
	Kunitz/Bovine pancreatic trypsin inhibitor Domain protein	1,274.39	0.07	0.02	0	DN56779
Autophagy-related protein	Autophagy-related protein 16	3.39	9.07	3.64	0.04	DN40035
	Autophagy-related protein 9A	7.65	9.52	6.28	0.32	DN55372
	Autophagy-related protein 12	24.88	35.90	22.76	0.13	DN37604

TPM values are the mean of the three biological replicates analyzed from each physiological condition. Significance analysis was based on one-way ANOVA (*P* < 0.05). Full information on unigene names, TPM data, and other information see Additional file 5: Table S5

In invertebrates, lipids perform several functions including serving as constituents of cellular structures, hormones, energy, and play roles in egg production and metamorphosis [56, 57]. Lipid metabolism is of crucial importance in *D. marginatus*, approximately 150 unigenes annotated were identified with a putative function in lipid metabolism. We selected 49 unigenes that are differentially expressed in blood-feeding and starvation (Table 3, Additional file 5: Table S5). The KEGG pathway enrichment revealed that sphingolipid metabolism, synthesis and degradation of ketone bodies, steroid hormone biosynthesis, arachidonic acid metabolism were related to lipid metabolism after blood-feeding. The unigene annotated as vitellogenin (DN57762) in the F tick group was significantly upregulated compared to both the H tick and M tick group. Vitellogenin is essential for egg development and oviposition, and has been shown to play a role in heme sequestration [58, 59]. In *D. variabilis*, it has been demonstrated that Vg expression increases after engorgement and Vg is exclusively expressed in fat body and gut cells of vitellogenic females but not in the ovary [60]. Beside vitellogenin, several apolipoproteins belong to the low-density lipoprotein family were upregulated. Corresponding to the lipoprotein, the low-density lipoprotein receptor (LDLR) was upregulated as well during blood-feeding, which is the major cholesterol-carrying lipoprotein of plasma, acting to regulate cholesterol homeostasis in cells [61]. In ticks, the LDLR binds LDL and transports it into digestive cells [62]. It has been considered that arthropods lack the set of enzymes to synthesize cholesterol, so they are obliged to obtain cholesterol from their hosts [63]. The unigenes involved in cholesterol metabolism were insulin induced protein (DN55975), high-density lipoprotein (DN55629), Niemann-Pick type C1 (DN34326) and Niemann-Pick type C2 (DN53284) during blood-feeding, and their expression profile indicated lipid metabolism was very active in newly molted ticks. In non-ruminants, the INSIG1 gene modulates cholesterol metabolism, lipogenesis, and glucose homeostasis with a cellular localization on endoplasmic reticulum membrane [64]. High density lipoprotein transports diglyceride from the fat body cell into the hemolymph, and is involved in the transport of cholesterol from the gut into the hemolymph in insects [65, 66]. Niemann-Pick type C1 encodes a large membrane glycoprotein with mostly a late endosomal localization. Niemann-Pick type C2 encodes a small soluble lysosomal protein with high affinity binding to cholesterol. Both proteins are in intracellular regulation of cholesterol metabolism, and their efficiency determine the processing and utilization of endocytosed cholesterol [67]. Many unigenes related to sphingolipid metabolism were upregulated after blood-feeding (Table 3). Sphingolipids are one of the major classes of eukaryotic lipids and were appreciated as components of the plasma membrane and as modulators of cell-cell interactions and cell recognition [68]. Other upregulated unigenes involved in lipid transport were the oxysterol-binding protein, hemelipoglycoprotein, fatty-acid binding protein, microsomal triglyceride transfer protein (Table 3), in which several were upregulated after long-term starvation. Unlike absorption of lipids during blood-feeding, the female adult ticks undergoing long-term starvation were in steady consumption of lipid reserves [18]. Compared to newly molted and engorged ticks, starved ticks presented most of the genes downregulated regarding lipid metabolism, whereas, a number of genes related to hormone synthesis and wax formation were upregulated in the H tick and M tick groups indicating these genes might play a role in survival of *D. marginatus* during the off-host period.

**Table 3:** Selected differentially expressed unigenes putatively involved in lipid metabolism after blood-feeding and long-term starvation

Protein/Function	Annotation	TPM F-tick	TPM H-tick	TPM M-tick	ANOVA (*P-*value)	Unigene
Lipoprotein	Vitellogenin	3,180.28	0.06	0.07	0	DN57762
	Vitellogenin-B	301.42	336.42	40.48	0	DN58267
	Low-density lipoprotein receptor-related protein	0.08	4.18	0.47	0	DN38842
	*Ixodes scapularis* hypothetical protein	46.51	19.10	4.59	0	DN58114
	Apolipoprotein	0.00	0.08	19.97	0	DN37679
	Apolipoprotein	0.00	1.52	41.34	0	DN34916
LDL-receptor	Lipophorin receptor, putative	43.28	20.04	9.58	0	DN55485
	LDL-receptor class A domain-containing protein	15.24	4.61	10.37	0	DN58047
	LDL-receptor class A domain-containing protein	18.39	0.29	3.81	0	DN50871
	LDLR chaperone boca-like	29.84	21.93	7.41	0.01	DN56139
Acyl-CoA synthetase	Acyl-CoA synthetase family member	33.65	2.18	0.51	0	DN42480
	Acyl-CoA synthetase family member	0.12	14.37	33.01	0	DN56802
Cholesterol metabolism	Insilin induced protein	8.99	7.13	13.76	0.02	DN55975
	Putative high-density lipoprotein-binding protein	103.71	19.57	4.93	0	DN55629
	Fatty acyl-CoA elongase	62.53	7.57	8.65	0	DN57046
	Fatty acyl-CoA elongase, putative	47.18	7.82	14.56	0	DN33724
	Fatty acyl-CoA elongase, putative	101.92	22.09	93.38	0.01	DN45177
Niemann-Pick type C1	Niemann-Pick type C1 domain-containing protein	43.49	19.39	11.53	0	DN50927
	Putative Niemann-Pick type C1 domain-containing protein	35.83	6.11	18.27	0	DN34654
	Putative Niemann-Pick type C1 domain-containing protein	176.11	0.29	0.03	0	DN34326
Niemann-Pick type C2	Major epididymal secretory protein HE1, putative	33.38	0.80	8.31	0	DN53284
Hemelipoglycoprotein	Hemelipoglycoprotein precursor	1,247.69	0.78	0.50	0	DN45062
	Hemelipoglycoprotein precursor	5.69	0.21	1.22	0	DN58109
Lipid transporter	Predicted microsomal triglyceride transfer protein	171.76	19.53	5.56	0	DN43048
	Fatty-acid and retinol-binding protein 1	0.00	6.56	0.00	0	DN59148
	Lipid droplet-associated hydrolase	0.20	9.14	0.45	0	DN48962
Oxysterol-binding protein	Oxysterol-binding protein-related protein 2	14.81	48.91	13.96	0	DN54120
	Oxysterol-binding protein-related protein 9	7.03	28.49	14.11	0.01	DN54199
Fatty acid-binding protein	Fatty acid-binding protein	1,056.66	724.66	357.63	0	DN48942
Glycerol kinase	Lycerol kinase-like isoform X2	6.08	11.88	2.60	0.01	DN47773
	Mitochondrial-like acylglycerol kinase	8.20	14.71	3.36	0.01	DN50098
Sphingolipid metabolism	Putative shingomyelin phosphodiesterase	17.53	0.13	0.13	0	DN45324
	Sphingomyelin phosphodiesterase 4-like isoform X1	6.38	22.13	8.08	0.02	DN55541
	Putative beta-glucocerebrosidase	114.00	0.05	0.26	0	DN35305
	Putative beta-glucocerebrosidase	494.63	0.21	0.05	0	DN53737
	Conserved hypothetical protein	5.51	8.20	1.59	0.01	DN56056
Ecdysone synthesis	Ecdysone-induced protein 75	4.98	35.95	13.26	0	DN40090
	Ecdysone-induced protein 63F1	1.51	16.91	6.71	0	DN38040
	Conserved hypothetical protein	7.24	0.11	0.02	0	DN38433
Phospholipase	Phospholipase A2 precursor, putative	36.94	0.08	0.13	0	DN31517
	Phospholipase A2 precursor, putative	194.59	29.04	41.49	0	DN56186
Gastric triacylglycerol lipase	Gastric triacylglycerol lipase, putative	9.9	0.31	0.82	0	DN47082
	Gastric triacylglycerol lipase, putative	26.2	7.34	0.93	0	DN52640
Cuticular wax formation	Acyl-CoA reductase, putative	2.68	2.25	400.44	0	DN56552
	Acyl-CoA reductase, putative	24.53	0.22	0	0	DN52493
	Acyl-CoA reductase, putative	23.75	0.03	0	0	DN43844
	Acyl-CoA reductase, putative	27.14	0.91	0.02	0	DN51418
	Acyl-CoA reductase, putative	47.33	1.04	0.87	0	DN43844
	Acyl-CoA reductase, putative	2.82	12.26	4.74	0	DN53780

TPM values are the mean of the three biological replicates analyzed from each physiological condition. Significance analysis was based on one-way ANOVA (*P* < 0.05). Full information on unigene names, TPM data, and other information see Additional file 5: Table S5

Carbohydrates, as proteins and lipids ingested with host blood, are the essential nutrients for ticks. A recent study demonstrated the major pathways involved in carbohydrate metabolism with details indicating blood glucose is an important nutrient for *I. scapularis* [20]. In the *D. marginatus* transcriptome analyzed in this study, several carbohydrases, carbohydrate transporters and cuticular proteins were identified (Table 4, Additional file 5: Table S5). Up to 55 unigenes on carbohydrate metabolism were differentially expressed (35 in the F tick group, 9 in the H tick group and 11 in the M tick group), covering biological processes such as carbohydrate phosphorylation, protein glycosylation, glycogen catabolic process, galactose metabolic process. The results indicated that the upregulated unigenes encoding carbohydrate-metabolizing enzymes were similar with the genes identified in the transcriptomes of *Ixodes ricinus* Linnaeus [17], *Haemaphysalis flava* Neumann [37], *D. variabilis* [69], and the argasids *Ornithodoros moubata* Murray [46] and *Ornithodoros erraticus* Neumann [63]. According to the annotations, the upregulated unigenes were associated with the metabolism and transport of several carbohydrates including glucose, fructose, mannose, galactose, maltose, idose, malate, and chitin (Table 4).

**Table 4:** Selected differentially expressed unigenes putatively involved in carbohydrate metabolism after blood-feeding and long-term starvation

Protein/Function	Annotation	TPM F-tick	TPM H-tick	TPM M-tick	ANOVA (*P-*value)	Unigene
Carbohydrate metabolism	Glycosyl hydralase, sucrase-isomaltase, putative	62.30	0.75	1.67	0	DN53826
	Alpha-L-fucosidase, putative	18.85	6.80	0.56	0	DN57768
	Beta-N-acetylhexosaminidase, putative	16.00	1.29	2.47	0	DN48906
	Beta-glucuronidase-like isoform X1	31.56	1.90	6.15	0	DN55789
	Poly (ADP-ribose) glycohydrolase-like isoform X3	3.15	8.91	4.75	0.06	DN48563
	Glucosidase II, putative	39.01	23.50	5.26	0	DN51547
	Glucose 6-phosphate dehydrogenase isoform C	22.75	66.04	34.30	0.02	DN56097
	Sucrase-isomaltase, intestinal-like	0.21	2.16	8.15	0	DN57119
	Alpha-L-iduronidase-like isoform X1	0.01	5.44	0.32	0	DN34070
	6-phosphofructo-2-kinase/fructose 2,6-bisphosphatase, putative	3.74	14.96	5.12	0.01	DN57996
Mannose metabolism	Mannosyl-oligosaccharide glucosidase, putative	45.53	14.34	5.08	0	DN34438
	Phosphomannomutase, putative	55.46	14.18	4.64	0	DN49511
	Mannose-1-phosphate guanyltransferase beta-like	99.60	39.29	10.32	0	DN47789
	Mannose-binding endoplasmic reticulum-golgi intermediate compartment lectin	160.77	41.80	8.51	0	DN36723
Galactose metabolism	Glycosyl hydralase, sucrase-isomaltase, putative	62.30	0.75	1.67	0	DN53826
	*Ixodes scapularis* Beta-galactosidase precursor, putative	37.86	5.82	0.40	0	DN53710
	Beta-1,4-N-acetylgalactosaminyltransferase bre-4-like	0.01	7.36	3.04	0	DN52083
	Galectin, putative	101.18	1,262.29	317.33	0	DN57588
Carbohydrate transport	UDP-galactose transporter, putative	8.55	21.71	8.43	0.13	DN52272
	Oxoglutarate/malate carrier protein, putative	6.33	44.22	6.32	0	DN57072
	Sodium-dependent glucose transporter, putative	0.84	7.65	0.36	0	DN43028
	Solute carrier family 2, facilitated glucose transporter Member 10-like	6.27	19.39	1.57	0	DN56465
	Sugar transporter, putative	1.99	12.64	1.20	0	DN52986
Chitin binding protein	Cuticular protein, putative	1.39	3.73	1,053.67	0	DN35205
	Cuticular protein, putative	0.10	27.47	113.61	0	DN57895
	Cuticular protein, putative	0.14	27.03	380.74	0	DN41881
	Peritrophin A, putative	33.01	4.28	250.60	0	DN52544
	Cuticular protein, putative	1.41	74.29	4.48	0	DN45546
	Hypothetical protein X975_15305	2.67	58.72	59.54	0	DN35800
	Salivary mucin	8.35	62.56	98.37	0	DN48751
	Conserved hypothetical protein	29.27	2.97	15.17	0	DN55376
	Peritrophin A, putative	43.35	2.96	17.71	0	DN44759
	Peritrophin A, putative	32.29	421.29	198.20	0	DN57787
	Hypothetical protein	130.84	26.98	80.95	0.03	DN46721
Cuticle protein	Structural constituent of cuticle, putative	0.04	0.09	1,571.25	0	DN43545
	Conserved hypothetical protein	0.01	0.05	133.71	0	DN54086
	Cuticle protein, putative	125.92	3.26	255.44	0	DN44745
	Conserved hypothetical protein	1.78	0.23	230.19	0	DN52842
	Peritrophic membrane chitin binding protein, putative	32.02	0.00	0.15	0	DN57209
	Calphotin-like	0.03	17.80	424.74	0	DN37939
	Structural constituent of cuticle, putative	0.00	0.78	271.20	0	DN35945
	Cuticle protein, putative	166.79	0.00	0.39	0	DN49371
	Cuticular protein, putative	2.28	0.18	268.89	0	DN55649
	Cuticle protein, putative	0.01	0.11	104.18	0	DN50628
	Cuticle protein, putative	4,618.14	3.97	5.64	0	DN49371
	Conserved hypothetical protein	1.78	0.23	230.19	0	DN52842
	Cuticular protein	0.87	391.58	1,332.75	0	DN49929
	Cuticle protein, putative	0.79	153.69	230.33	0	DN39452
	Cuticular protein, putative	11.83	0.19	40.79	0	DN51261
	Cuticle protein, putative	125.92	3.26	255.44	0	DN44745
	Cuticular protein, putative	0.22	209.83	68.04	0	DN55323
	Cuticular protein, putative	2.36	2.31	39.73	0	DN52617
	Cuticular protein-like protein	7.12	2.89	55.46	0	DN54351
	Cuticle protein	3,195.63	128.25	2,513.73	0	DN46667

TPM values are the mean of the three biological replicates analyzed from each physiological condition. Significance analysis was based on one-way ANOVA (*P* < 0.05). Full information on unigene names, TPM data, and other information see Additional file 5: Table S5

In the F tick group, most upregulated unigenes on carbohydrate metabolism were glycosyl hydrolase (DN53826), mannose-binding lectin (DN36723), beta-galactosidase (DN53710), and α-L-fucosidase (DN57768). Glycosyl hydrolase is a kind of enzyme that hydrolyzes glycosidic bonds, and plays an important role in the hydrolysis and synthesis of glycoconjugates and glycosidic compounds [70]. In invertebrate mannose-binding lectin was related to specific pattern recognition of the complement system *via* activation of the lectin pathway [71, 72]. β-galactosidase is widely found in various animals, plants and microorganisms that hydrolyses the β-glycosidic bond formed between a galactose and its organic moiety [20, 63]. The data on a digestive α-L-fucosidase activity of *Amblyomma cajennense* Fabricius were described in a study and a plausible inference was present that the upregulation of the gene was probably related to removal of fucose produced by microorganisms in tick gut [73, 74]. In our study, the α-L-fucosidase was over-expressed both in the F tick and H tick group.

In the H tick group, most upregulated unigenes on carbohydrate metabolism were galectin (DN57588), glucose 6-phosphate dehydrogenase (DN56097) and 6-phosphofructo-2-kinase/fructose 2,6-bisphosphatase (DN57996). Galectins are a glycan-binding superfamily protein (lectins) that plays an important role in insect and tick development, interaction with pathogens, and the innate immune system by recognizing repeating saccharide units found on microbial surface glycoproteins in immunity, respectively [75, 76]. Glucose 6-phosphate dehydrogenase (G6PDH) is a NADPH-producing enzyme that was of critical role in the oxidative stage of the pentose phosphate pathway [77]. A study on *R. microplus* G6PDH showed four transcripts differentially expressed in engorged and/or unfed adults in which G6PDH-D showed upregulation as the one found in this study [78]. The 6-phosphofructo-2-kinase/fructose 2, 6-bisphosphatase (PFK-2/FBase-2) is a bifunctional enzyme that catalyzes either the synthesis (PFK-2 activity) or the degradation (FBase-2 activity) of fructose-2, 6-bisphosphate [79]. The results revealed that upregulation of PFK-2/FBase-2 in H tick after long-term starvation might be related to fructose-2, 6-bisphosphate.

In the M tick group, most of the unigenes related to carbohydrate as energy were not significantly upregulated, while unigenes on chitin synthesis and binding were significantly upregulated (Table 4). In the transcriptome of *D. marginatus*, up to 30 unigenes were matched with PF00379 domain and another 30 unigenes were matched with PF01607 in the Pfam database. These two chitin binding domains were generally found in arthropods [80]. The majority of them annotated as structural constituent of cuticle, peritrophic membrane chitin binding protein, and conserved hypothetical protein were upregulated in the M tick group, and the highly expressed unigenes were cuticular proteins indicating that *D. marginatus* female adult shortly after completion of metamorphism were highly expressed, while the functional annotations of these proteins were inadequate. The chitin binding peritrophic-A domain (PF01607: CBM14) may be involved in formation and maintenance of peritrophic membrane [81] (Table 4, Additional file 5: Table S5). The peritrophic membrane is considered having the function of protecting microvilli of midgut epithelial cells from mechanical damage, pathogens and toxic substances, and acts as a semipermeable barrier allowing transport of small molecules and nutrients [82]. The structure of tick peritrophic membrane has been described in *I. scapularis* [83], *I. ricinus* [84], *Haemaphysalis longicornis* Neumann [85] and *Ixodes dammini* Spielman [86]. The chitin-binding cuticular protein (PF00379) includes an amino acid motif which functions to bind chitin, but the protein shows no sequence similarity to the known chitin-binding domain (PF01607: CBM14) found in chitinases and some peritrophic membrane proteins [80]. Cuticular protein comprises highly organized structural products that is formed as a layered and extracellularly secreted protein from the epidermis of insects [87]. The upregulated unigenes encoding cuticular proteins in newly molted ticks could be related to sclerotization and melanin formation of the tick surface cuticle, as a previous report has described at length that the two process may occur in concert in insects [88].

Ticks own effective defense mechanisms to protect from pathogenic microorganisms and to maintain the intestinal microbiota at a tolerable level [89]. In the *D. marginatus* transcriptome, 13 upregulated unigenes were among 27 unigenes annotated with immune functions putatively encoding defensins, lysozyme, alpha-macroglobulin, and hemolectin (Table 5, Additional file 5: Table S5). Regarding defensins, there were upregulations shown in all three states of *D. marginatus* transcriptome data. The most highly expressed defensin was DN36307 (TPM: 2888.17) in the H tick group. Defensin is an antibacterial peptide that is the major components of innate immunity in ticks and protect ticks from gram-negative, gram-positive bacteria, and plasmodium [90–92]. Lysozymes are effective peptides which play a role in carbohydrate digestion suppressing the proliferation of Gram-negative bacteria in the midgut [93]. Alpha-macroglobulin plays an important role in phagocytosis of microbes, and may specifically be involved in phagocytosis of a certain genus of microbes in ticks [94]. Several α-macroglobulins were upregulated in the F tick group after blood-feeding indicating the ticks were trying to control microbial populations in the system. Besides α-macroglobulins, a hemolectin was upregulated in F tick group which is a major clotting factor in insects [95, 96], and also has a function help insect immune system against bacterial infection [97].

**Table 5:** Selected differentially expressed unigenes putatively involved in defense response and antioxidant response after blood-feeding and long-term starvation

Protein/Function	Annotation	TPM F-tick	TPM H-tick	TPM M-tick	ANOVA (*P-*value)	Unigene
Defensin	Hebreain	404.98	10.32	32.75	0	DN38627
	Hebreain	352.36	2,888.17	618.80	0	DN36307
	Antimicrobial peptide microplusin	8.32	73.65	178.51	0	DN42974
	Defensin	21.66	14.95	124.77	0	DN37748
	Putative defense protein 3-like protein	76.62	8.28	2.10	0	DN33990
	Putative defense protein 1	8.27	1.99	123.46	0	DN44863
	Conserved hypothetical protein	0.61	0.21	76.55	0	DN51960
Alpha-macroglobulin	Alpha-macroglobulin, putative	80.00	9.26	5.26	0	DN48632
	Alpha-macroglobulin, putative	28.02	0.98	3.19	0	DN55988
Lysozyme	Lysozyme	30.22	104.76	10.41	0	DN43837
	Lysozyme, putative	24.63	18.74	51.02	0.01	DN54894
	Gamma-interferon inducible lysosomal thiol reductase	1.89	25.10	1.06	0	DN37451
Hemolectin	Hemolectin, putative	9.64	1.61	0.20	0	DN58201
Glutathion S-transferase	Putative glutathione S-transferase	54.51	4.40	0.45	0	DN41472
	Glutathione S-transferase	377.46	61.22	21.56	0	DN45728
	Glutathione S-transferase, putative	76.98	12.08	12.37	0	DN49881
	Glutathione S-transferase, putative	63.43	9.89	9.71	0	DN40498
	Putative glutathione S-transferase	103.27	2.50	0.21	0	DN29689
	Glutathione S-transferase, putative	141.94	17.66	43.40	0	DN39983
	Glutathione S-transferase, putative	83.97	50.83	18.84	0	DN51596
	Glutathione S-transferase, putative	34.12	23.17	3.01	0	DN46189
	Glutathione S-transferase, putative	24.53	3.86	15.36	0	DN48615
	Glutathione S-transferase, putative	4.67	2.92	11.30	0	DN51816
	Glutathione S-transferase	38.31	40.50	80.58	0	DN50592
	Putative glutathione S-transferase	145.21	23.61	6.79	0.01	DN38169
	Putative glutathione S-transferase	2,285.92	1,869.90	234.28	0.01	DN56344
	Glutathione S-transferase	17.36	2.77	11.28	0.01	DN50142
	Glutathione S-transferase, putative	70.00	33.69	22.33	0.01	DN44573
	Microsomal glutathione S-transferase	44.68	67.61	24.03	0.03	DN40419
Antioxidant activity	Catalase	624.90	207.05	171.21	0	DN57352
	Protein disulfide isomerase-2	408.68	45.01	22.40	0	DN50839
	Protein disulfide isomerase	781.58	202.00	98.63	0	DN52494
	Thiol-disulfide isomerase, putative	41.52	14.07	2.77	0	DN46036
	Protein disulfide isomerase-1	95.20	34.10	14.70	0	DN50839
	Thioredoxin peroxidase	186.76	29.83	10.68	0	DN42520
	16 kDa thioredoxion, putative	155.43	66.01	29.23	0	DN37149
	Glutathione peroxidase	1,434.27	174.50	46.00	0	DN40806
Iron metabolism	Ferritin 2	78.53	84.06	22.75	0.01	DN51538
	Transferrin receptor, putative	80.18	0.03	0.03	0	DN54992
Superoxide dismutase	Hypothetical protein	16.17	0.75	0.49	0	DN50617
	Superoxide dismutase Cu-Zn, putative	17.30	5.66	7.58	0	DN52749
	Unnamed protein product	22.05	38.38	96.10	0	DN40853
	Superoxide dismutase [Cu-Zn] isoform X2	10.76	2.49	6.86	0	DN41589
Heat shock proteins	Heat shock proteins 90, putative	312.01	72.53	29.59	0	DN49937
	Heat shock 70 kDa protein	287.74	81.31	31.94	0	DN38789
	Heat shock 70 kDa protein	341.26	225.29	70.48	0	DN48195
	Heat shock protein 70	1.54	10.26	9.86	0	DN48944
	Heat shock proteins 60	51.74	106.17	19.96	0	DN55315
	Mitochondrial chaperonin hsp60	2.19	18.04	0.13	0	DN33794
	Heat shock HSP20 protein, putative	5.00	40.29	7.56	0	DN44693
	Putative heat shock-related protein	98.50	1.11	2.96	0	DN43840
	Stress-induced protein 1	0.00	17.55	0.00	0	DN58874
	PREDICTED: heat shock protein 16-like	0.75	19.81	0.14	0	DN37163
	Small heat shock protein I	829.95	124.30	124.53	0	DN45806
	Small heat shock protein I	860.03	112.86	55.58	0	DN46736
	Putative heat shock-related protein	79.00	6.59	6.37	0	DN45729
	Small heat shock protein II	13.34	61.93	28.28	0	DN46199
	Heat shock protein, putative	92.50	174.46	24.35	0	DN49326

TPM values are the mean of the three biological replicates analyzed from each physiological condition. Significance analysis was based on one-way ANOVA (*P* < 0.05). Full information on unigene names, TPM data, and other information see Additional file 5: Table S5

Ingestion of large amounts of host blood and the digestion of the blood meal increases redox pressure in ticks. Therefore, detoxifying mechanisms are required for counteracting redox pressure in ticks [98]. There were 64 upregulated transcripts coding for proteins with oxidoreductase activities, chaperone proteins, and proteins involved in iron and hemoglobin metabolism (Table 5). Most of the upregulated unigenes were in the F tick group. In the differentially expressed unigenes, a glutathione S-transferase (GST) gene (DN56344) was highly expressed both in F tick (TPM: 2,285.92) and H tick (TPM: 1,869.90) groups. GSTs are known as genes of a superfamily that are involved in the detoxification of endogenous and xenobiotic compounds [99]. Blood-feeding increased cellular stresses, and the majority of the GSTs were upregulated except two omega class GSTs (GSTO) that were upregulated in the M tick group (DN51816; DN50592). The GSTOs were characterized in insects, and are over-express under stress response and at the presence of insecticides [100, 101]. Other highly expressed antioxidant proteins included catalase (DN57352; TPM: 624.90), protein disulfide isomerase (DN52494; TPM: 781.58) and glutathione peroxidase (DN40806; TPM: 1,434.27).

Heat-shock proteins (HSP) work as molecules involved in correction of protein folding which were over-expressed under heat shock and other stress responses as ticks ingesting host blood with elevated temperature, dealing with environmental stress, and neutralizing damage caused by toxic substances ingested with blood meal or produced during digestion of blood meal [102, 103]. We selected 16 differentially expressed HSPs including HSP90, HSP70, HSP60 and HSP20 (small HSPs) (Table 5). After blood-feeding, The HSP90 (DN49937; TPM: 312.01) over-expressed in the F tick group which was 5.8 folds the expression of the gene in the H tick group and 3 folds the expression of the gene in the M tick group. Notably a HSP20 (DN58874) was exclusively expressed in the H tick group. Two highly differentially expressed HSP70s (DN38789 and DN48195) were in the F tick group. Significantly upregulated and highly expressed HSP20s were also found in the F tick group implying upregulation of these genes may induced by feeding and blood-meal digestion [46], while upregulation of unigenes including HSP60 (DN55315 and DN33794) and HSP20 (DN49326, DN46199 and DN44693) in the H tick group were likely caused by exhaustion of energy substance and environmental stress after long-term starvation [104, 105].

Ticks acquire iron and heme from host blood, while ticks can not utilize heme for bioavailable iron [106]. In the iron metabolism ticks would have to bear redox pressure from acquiring iron from host transferrin [28]. In the transcriptome of *D. marginaus*, two unigenes annotated on iron metabolism were significantly upregulated in the F tick group including ferritin 2 (DN51538) and transferrin receptor (DN54992) (Table 5). Tick ferritin 2 is a unique secreted protein that is conserved in ticks [30], and tick ferritin 2 is a gut-specific protein which is secreted into the hemolymph transporting bioavailable iron in between organs [107]. The iron in host transferrin was released by lysosome in tick midgut through endocytosis that was triggered when transferrin receptor conjugated with host transferrin [106, 108], but further detailed research is needed to characterize molecular function of tick transferrin receptors. Generally, feeding and digestion of haemoglobin in blood meal releases large amounts of heme and subsequently cause the upregulation of antioxidant genes, whereas exhaustion of energy reserve would increase reactive oxygen species, which in turn would exacerbate starvation-induced autophagy [109].

### RT-qPCR validation of RNA-seq data

**Fig. 7:**
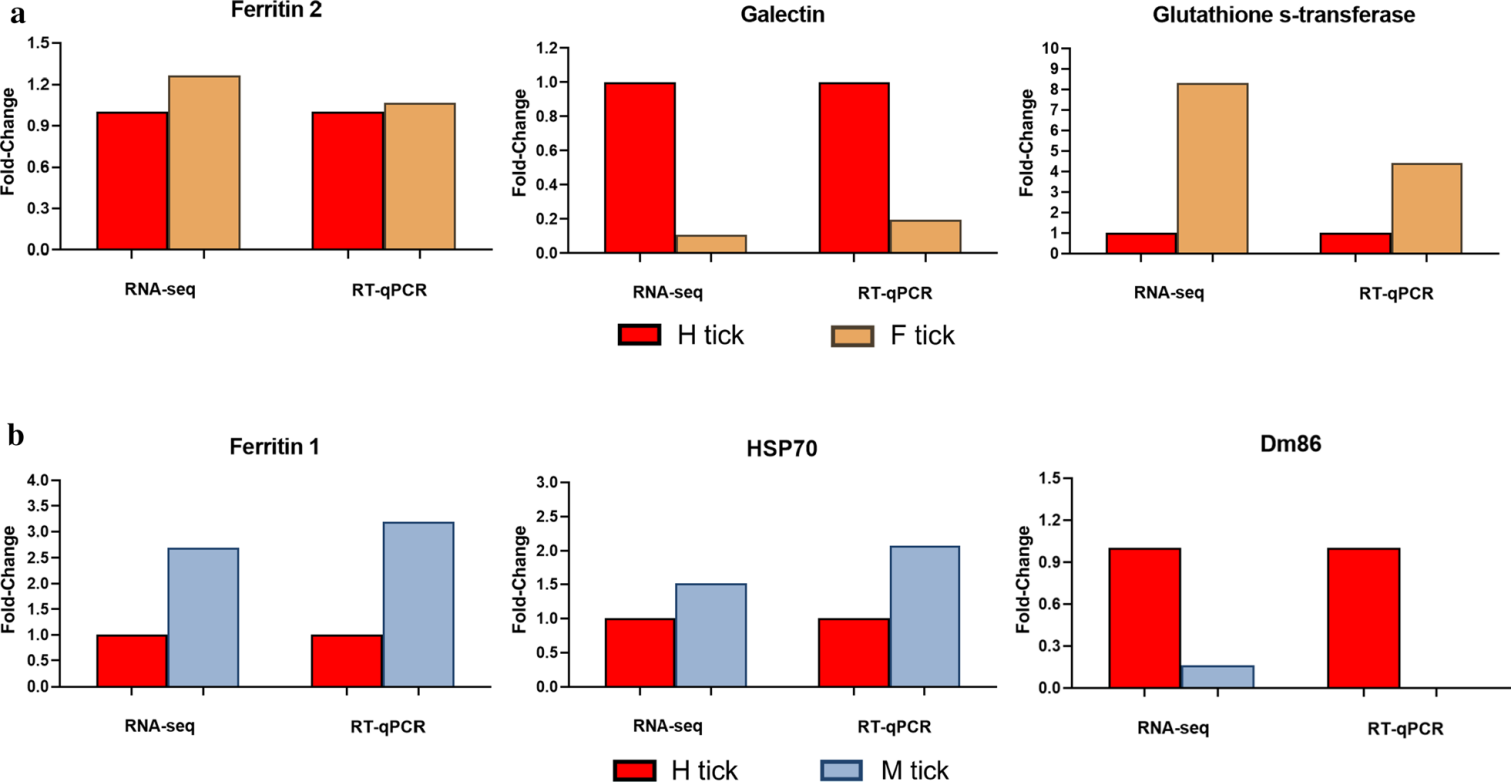
RT-qPCR validation of selected 6 differentially expressed genes in the RNA-seq. RT-qPCR was performed using cDNA templates derived from female *D. marginatus* ticks. The expression profiles of the 6 genes in 3 different status of the tick are indicated above. Red bar represents starved female ticks (H tick), beige bar represents fed female ticks (F tick), and blue bar represents newly molted female ticks (M tick). The expression level of each gene was normalized to its level in starved ticks

In order to validate RNA-seq results, the expression level of six selected upregulated genes were assessed by RT-qPCR, using *ef-1α* (DN43634) as a reference. The selected genes encoded the following proteins: ferritin 1; ferritin 2; HSP70; galectin; GST; and Dm86 (Fig. 7). For checking the suitability of the primer pairs, the amplification products were electrophoresed in an agarose gel, and the density of the bands of expected size were measured for all genes, including the housekeeping genes. The comparison between the RT-qPCR and RNA-seq results confirmed that all genes showed a similar trend in transcriptional expression changes, the normalized fold change values of all unigenes by *ef-1α* (DN43634) were recorded in Additional file 5: Table S5. Transcripts encoding ferritin 1, HSP70, GST, galectin and Dm86 showed rather similar expression patterns, while, ferritin 2 did not show differential expression in RT-qPCR (Fig. 7). The less consistent result obtained from RT-qPCR and RNA-seq may be due to several reasons including variability in the expression of the reference gene in different biological samples or differences in data standardization method between RT-qPCR and RNA-Seq analyses [37].

The biological significance of the gene expression profiles is important, but the gene expression alone could not characterize them, as further investigations are required. In total, the differences among the gene expression profiles indicate that the transcriptome of *D. marginatus* female ticks are informative, which may provide data for further research on this tick species.

## Conclusions

In this study we expanded sequence information of *D. marginatus* by RNA-seq data and provided functional annotations of the genes of this tick species on a whole-body transcriptome level. We found that blood-feeding and starvation induced a strong upregulation of unigenes associated with proteins, lipids and carbohydrates metabolism, as well as unigenes associated with defense mechanism and antioxidant activity. Overall, these results would facilitate the understanding of *D. marginatus* genes encoding potential target molecules for efficient anti-tick interventions.

## Supplementary information

**Additional file 1: Table S1.** Specific primers used in the RT-qPCR validation of RNA-seq results.

**Additional file 2: Table S2.** Overview of sequencing quality control.

**Additional file 3: Table S3.** Annotation summary with DIAMOND software.

**Additional file 4: Table S4.** Raw reads alignment results.

**Additional file 5: Table S5**. Annotation table of *D. marginatus* female adult transcriptome analysis.

**Additional file 6: Table S6.** Unigenes used in molecular function, cellular component and biological process anaylisis by GO annotation.

**Additional file 7: Figure S1.** Genes differentially expressed between groups after blood-feeding and long-term starvation.

## Data Availability

The datasets supporting the conclusions of this article are available in the GEO database of GenBank repository (https://www.ncbi.nlm.nih.gov/geo/query/acc.cgi?acc=GSE151667) with accession number GSE151667.

## Abbreviations

RNA-seq: RNA sequencing
TPM: transcripts per million
DEGs: differentially expressed genes
RT-qPCR: real-time quantitative PCR
GEO: Gene Expression Omnibus database
GO: Gene Ontology database
KEGG: Kyoto Encyclopedia of Genes and Genomes database
