# Supplementary material for: De novo assembly and analysis of the transcriptome of the Dermacentor marginatus genes differentially expressed after blood-feeding and long-term starvation
Source: Parasit Vectors. 2020 Nov 10;13:563. doi: 10.1186/s13071-020-04442-2 (PMC7654163; doi:10.1186/s13071-020-04442-2)
Supplement: Supplementary file 1 — Additional file 1: Table S1. Specific primers used in the RT-qPCR validation of RNA-seq results. [file 13071_2020_4442_MOESM1_ESM.docx]

**Table S1** Specific primers used in the RT-qPCR validation of RNA-seq results

| **Primer pairs** | **(5’ to 3’ end)** | **Anotation** | **Unigene** | **PCR Product** |
| --- | --- | --- | --- | --- |
| Fer1-RT-F  Fer1-RT-R | CCAGAAGCCAGCACAGGAC  CCTTGATGGACTTCACCTGC | Ferritin 1 | DN38021 | 185 |
| Fer2-RT-F  Fer2-RT-F | CTTCCTCCACGACCGATGT  GCTCCTCGCTGGACTGGT | Ferritin 2 | DN51538 | 158 |
| HSP70-RT-F  HSP70-RT-R | CGCAACACAACCATTCCCAC  CCTTGGACAGGCGACCTTTAT | HSP70 | DN48195 | 273 |
| Galectin-RT-F  Galectin-RT-R | GGAACCGCCCAAGCAAG  CGCCGGAGATGATGACCAC | Galectin | DN57588 | 167 |
| GST-RT-F  GST-RT-R | TAGCGTTCCCGCAAGT  CAGGTATTCCTCGGTGAG | Glutathione S-transferase | DN38169 | 292 |
| Dm86-RT-F  Dm86-RT-R | ACCAGCAACAAACGAGG  GGTCCAGGATCTGAACAAC | Dm86 | DN57937 | 165 |
| Ef-1α-RT-F  Ef-1α-RT-R | CCGCCCAGGTTATTGT  ACGACGGTCGCACTTT | Elongation factor 1 alpha | DN43634 | 125 |
