# Supplementary material for: De novo assembly and analysis of the transcriptome of the Dermacentor marginatus genes differentially expressed after blood-feeding and long-term starvation
Source: Parasit Vectors. 2020 Nov 10;13:563. doi: 10.1186/s13071-020-04442-2 (PMC7654163; doi:10.1186/s13071-020-04442-2)
Supplement: Supplementary file 3 — Additional file 3: Table S3. Annotation summary with DIAMOND software. [file 13071_2020_4442_MOESM3_ESM.docx]

**Table S3** Annotation summary with DIAMOND software

| **Database** | **Number** | **Ratio (%)** |
| --- | --- | --- |
| All | 30,251 | 100.00 |
| GO | 7,527 | 24.88 |
| KEGG | 7,081 | 23.41 |
| Pfam | 7,663 | 25.33 |
| Swissprot | 6,825 | 22.56 |
| EggNOG | 9,315 | 30.79 |
| NR | 9,701 | 32.07 |
