# Supplementary figures and images for: De novo assembly and analysis of the transcriptome of the Dermacentor marginatus genes differentially expressed after blood-feeding and long-term starvation
Source: Parasit Vectors. 2020 Nov 10;13:563. doi: 10.1186/s13071-020-04442-2 (PMC7654163; doi:10.1186/s13071-020-04442-2)

# Differentially expressed genes in different groups

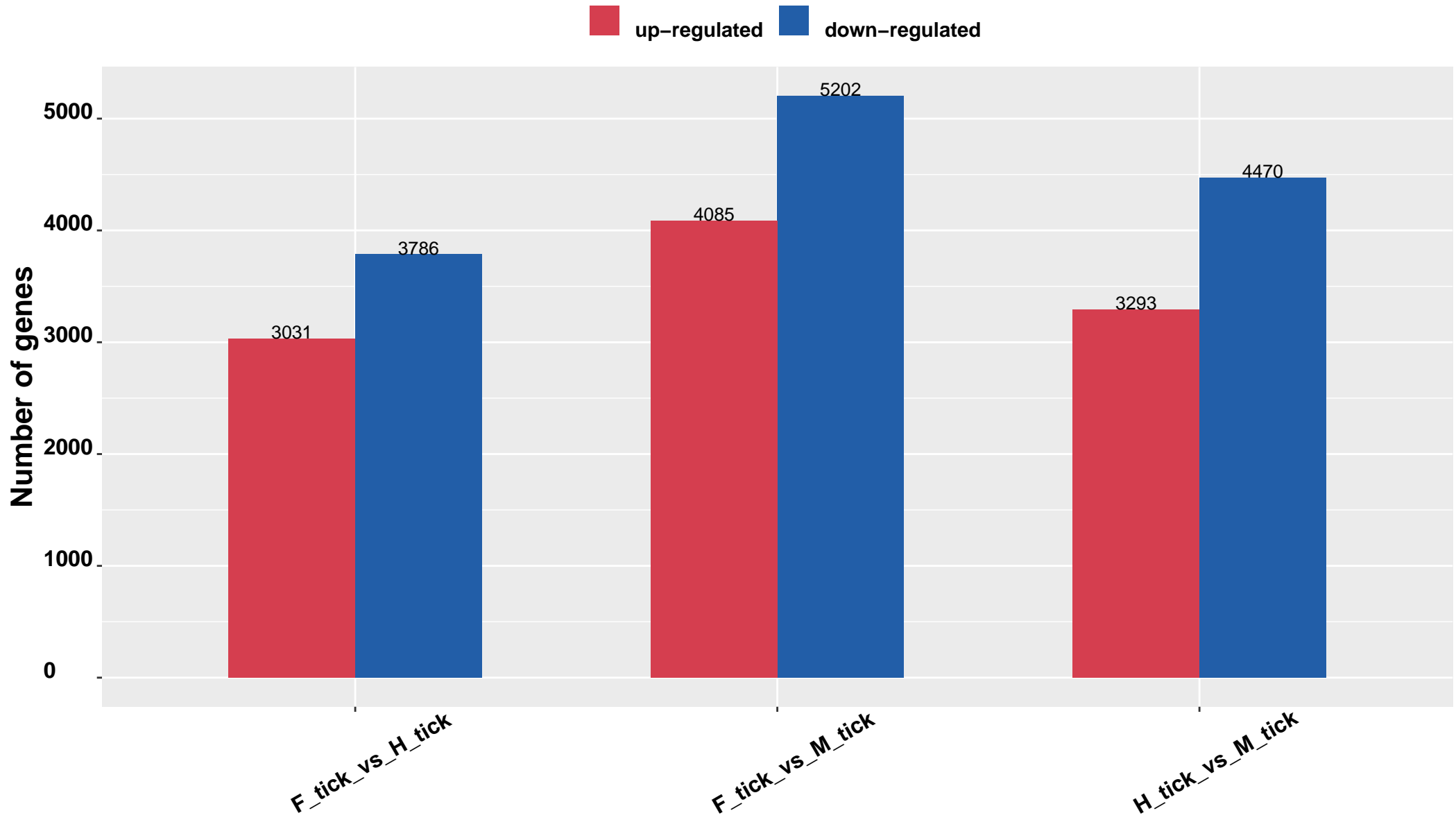

Supplement: Supplementary file 7 — Additional file 7: Figure S1. Genes differentially expressed between groups after blood-feeding and long-term starvation. [file 13071_2020_4442_MOESM7_ESM.pdf]
